# Supplementary material for: Spatial transcriptomics maps host–gut microbiome biogeography at high resolution
Source: Nat Microbiol. 2026 Mar 6;11(5):1193–204. doi: 10.1038/s41564-026-02286-7 (PMC13171632; doi:10.1038/s41564-026-02286-7)
Supplement: Supplementary file 2 — Reporting Summary [file 41564_2026_2286_MOESM2_ESM.pdf]

nature portfolio

Corresponding author(s): Iwijn De Vlaminck

Last updated by author(s): 01/21/2026

Reporting Summary

Nature Portfolio wishes to improve the reproducibility of the work that we publish. This form provides structure for consistency and transparency in reporting. For further information on Nature Portfolio policies, see our Editorial Policies and the Editorial Policy Checklist.

Statistics

For all statistical analyses, confirm that the following items are present in the figure legend, table legend, main text, or Methods section.

n/a	Confirmed	
☐	☒	The exact sample size (*n*) for each experimental group/condition, given as a discrete number and unit of measurement
☒	☐	A statement on whether measurements were taken from distinct samples or whether the same sample was measured repeatedly
☒	☐	The statistical test(s) used AND whether they are one- or two-sided<br>*Only common tests should be described solely by name; describe more complex techniques in the Methods section.*
☒	☐	A description of all covariates tested
☒	☐	A description of any assumptions or corrections, such as tests of normality and adjustment for multiple comparisons
☒	☐	A full description of the statistical parameters including central tendency (e.g. means) or other basic estimates (e.g. regression coefficient) AND variation (e.g. standard deviation) or associated estimates of uncertainty (e.g. confidence intervals)
☒	☐	For null hypothesis testing, the test statistic (e.g. *F*, *t*, *r*) with confidence intervals, effect sizes, degrees of freedom and *P* value noted<br>*Give P values as exact values whenever suitable.*
☒	☐	For Bayesian analysis, information on the choice of priors and Markov chain Monte Carlo settings
☒	☐	For hierarchical and complex designs, identification of the appropriate level for tests and full reporting of outcomes
☒	☐	Estimates of effect sizes (e.g. Cohen's *d*, Pearson's *r*), indicating how they were calculated

Our web collection on statistics for biologists contains articles on many of the points above.

Software and code

Policy information about availability of computer code

Data collection

No software was used to collect data in this study.

Data analysis

Preprocessing and alignment of spatial transcriptomics data
To ensure similar alignment and quantification across platforms and methodologies we used the "slide_snake" pipeline that utilizes Snakemake53 (6.1.0), which can be found on github (https://github.com/mckellardw/slide_snake). For the Visium and STRS (Visium) libraries, the pipeline first trims poly(A) and poly(G) sequences, as well as primer sequences using cutadapt54. The reads were aligned using STAR v2.7.10a55 and STARSolo56 (specified parameters: --outFilterMultimapNmax 50, --soloMultiMappers EM, --clipAdapterType CellRanger4) to generate expression matrices for every sample. For downstream analyses the GeneFull matrices were used. Barcode whitelists and the associated spot spatial locations for Visium data were copied from the Space Ranger software ("Visium-v1_coordinates.txt"). For the StereoSeq and STRS (StereoSeq) libraries, barcode maps were provided by the manufacturer as .h5 files and converted to text format using ST_BarcodeMap (https://github.com/STOmics/ST_BarcodeMap). Alignment references were generated from the GRCm39 reference sequence using GENCODE M32 annotations.

Unmapped reads classification and construction of microbiome Anndata objects
In this study, to classify reads of microbial origin out of the unmapped reads we utilized Kraken2 (version 2.09)21. We used the standard Kraken2 database supplemented with the mouse genome. Unmapped reads flagged in the BAM file were processed to retain the correct cell barcode and unique molecular identifier (UMI) information as identified by STARsolo. This allowed for the demultiplexing of Kraken2 output by cell barcode and UMI. For data integration, we employed Pandas, Scanpy, NumPy, Scipy, and regular expressions to create an AnnData object with cell barcodes as observations and NCBI taxonomy IDs as features. Only classified reads were retained for subsequent analysis.

Image Registration and Cell Segmentation

For Visium and Visium+PAP samples, image registration was performed using the 10x Genomics Loupe Browser. Hematoxylin and eosin (H&E)-stained tissue images were aligned to the spatial capture array, and regions corresponding to tissue and lumen were manually annotated. For Stereo-seq samples, nuclear-stained fluorescence images were acquired during sample processing. Image registration and cell segmentation were carried out using the Stereo-seq Analysis Workflow (SAW) provided by STOmics. Fluorescence images were aligned to the chip layout based on the ChipID metadata. Following registration, automated cell segmentation was performed using SAW's built-in algorithms. Segmentation masks were used to define cell boundaries, and barcodes within each segmented region were aggregated to construct single-cell transcriptomes. These cell-level datasets were then used for downstream spatial analysis and deconvolution.

Processing and Alignment of Metatranscriptomic Libraries
Metatranscriptomic sequencing data were processed using a custom computational workflow. Adapter trimming and quality filtering were performed using BBDuk (v38.90). Filtered reads were aligned to the mouse genome (GRCm39) with STAR (v2.7.10a) using GENCODE M32 annotations. Gene-level quantification was carried out with featureCounts (v2.0.0). Duplicate reads were identified and marked using Picard MarkDuplicates (v2.19.2). Reads that did not align to the host genome were extracted and taxonomically classified using Kraken2 (v2.0.9).

Sterile control pre-processing and identification of taxa to filter
To assess the Kraken2 classified microbial counts occurring in non-intestinal tissues for the low-resolution platform we re-aligned previously published Visium and STRS libraries of mock-infected C57BL/6J 11 days year old mice with and without polyadenylation as described in the corresponding studies14,20. 85 taxa occurring at 1ppm (UMI) or greater were excluded from downstream analysis as potential misclassification. For the Stereo-seq libraries, a sterile control experiment was conducted. Briefly, fresh-frozen heart from a eleven day old mouse were sectioned on a Stereo-seq 1cm x 1cm tile (STOmics, BGI). The sample was fixed in methanol at -20°C for 20 minutes followed by the in situ polyadenylation and the Stereo-seq library preparation protocol as described above. Taxa occurring at frequencies higher than 1 ppm UMI were excluded from downstream analyses.

Pre-processing of the Visium and Visium + PAP data
Spatial coordinates were assigned to the Visium and Visium + PAP library spots based on the barcode map provided by the Space Ranger software ("Visium-v1_coordinates.txt"). The accompanying hematoxylin and eosin histology images of each experiment were used to manually mark the spots that correspond to tissue and lumen. Scanpy57, mudata58,59, and muon58 were used to construct multimodal objects separately for the microbial maps (in the taxonomic levels of phylum, family, genus, and species). This was done for each one of the accounted microbial superkingdoms of Archaea, Bacteria and Viruses. For downstream analyses, only the spots covered by tissue or corresponding to lumen were accounted for.

Microbial percentage and enrichment calculation for the paired Visium and Visium + PAP experiments
For the three discussed superkingdoms, the percentage of reads falling under to a superkingdom classification was calculated as the percentage of Kraken-classified reads that belong to the superkingdom over the total counts of the library defined as the sum of unique molecules aligned to the host and unique molecules classified by Kraken2. The enrichment for each paired experiment was defined as the ratio of those percentages.

Relative abundance and bacterial richness calculations for the low-resolution datasets
To calculate the relative abundance for each examined sample, at family level, the corresponding family reads were collapsed and divided by the total molecules originating from bacteria as classified by Kraken2. The microbial richness per spot was calculated as the number of unique taxa occurring per spot after the exclusion of taxa accounting for 0.01% or less of microbial molecules in the whole sample. For the transverse axis relative abundance analysis, cells were spatially binned from the tissue to the lumen based on their minimum distance to the lumen-associated region. Phyla relative abundance data were then aggregated within each bin to quantify relative abundances across the tissue-lumen axis.

Rarefaction and Sequencing Saturation Analysis
Rarefaction analysis was performed on paired Visium and Visium+PAP libraries collected from four intestinal regions (proximal small intestine, ileum, cecum, and colon). Sequencing data were subsampled at defined fractions (10% to 100% of total reads). After alignment to the mouse genome using the Snakemake-based pipeline, unmapped reads were extracted and taxonomically classified using Kraken2. AnnData objects were generated at multiple taxonomic levels as described for the full dataset. For classified microbial reads in each condition, rarefaction curves were generated by plotting the number of unique bacterial molecules against sequencing depth. Michaelis-Menten models were fitted using non-linear least squares to estimate theoretical saturation behavior, and model fit was evaluated using the coefficient of determination (R^2). To calculate saturation for the total library, as well as for host- and microbiome-derived reads, the following general formula was used:

Here, modality refers to the source of the molecules: host-aligned reads, microbial reads classified by Kraken2, or all reads combined. This metric captures diminishing returns in the recovery of unique molecules with increasing sequencing depth. Saturation was computed at each subsampling level and modeled using Michaelis-Menten kinetics constrained to a maximum of 1. The resulting curves were used to estimate the sequencing depth required to achieve saturation (e.g., Saturation ≈ 0.9).

Gram stain comparison of Visium + PAP to the bulk RNA measurement
Adjacent samples profiled by Visium+PAP and by bulk metatranscriptomics were compared at the genus level. Reads were taxonomically classified with Kraken2 and counts were aggregated by genus. The same taxa excluded in spatial control analyses were removed prior to comparison. Gram-stain labels for genera were retrieved from BacDive60 and were curated to fill missing entries for highly abundant genera. The curated set included the Gram-negative: Pseudoprevotella, Hoylesella, Segatella, Allomuricauda, Marvinbryantia, Lachnoclostridium, Vescimonas, Mediterraneibacter, Coprococcus, Caproicibacterium, Massilistercora, Tellurirhabdus, Blattabacterium, Koleobacter, and Faecalitalea. The labels were used to calculate percent Gram-positive, Gram-negative, unknown or unclassified for the two types of measurement.

Cell type deconvolution
We employed the cell2location27 model (version 0.1.3) to deconvolve spatial transcriptomics data for the experiments conducted with both Visium and Stereo-seq technologies. The scRNA-seq reference, derived from a previous study on Apc Min/+ mice26 was filtered to include only genes that are highly expressed and informative for identifying rare cell types, with thresholds set at cell_count_cutoff = 5, cell_percent_cutoff = 0.01, and nonz_mean_cutoff = 1.12. Cell-type-specific expression signatures were generated using negative binomial regression from these selected genes. These signatures were applied to the spatial transcriptomics data to determine cell-type identities, with

the highest prediction scores used for assignment. For Visium, we set N_cells_per_location to 30, and for Stereo-seq, we set it to 1, with the detection_alpha parameter set to 20 in both cases.

Bacterial gene function analysis
Bacterial reference resources comprised selected abundant bacterial genomes (accession IDs: GCF_037113525.1, GCF_025148285.1) and bacterial genes downloaded from NCBI (downloaded 2025-09-18); for the genes, we retrieved and used all sequences annotated under the names atpA, enolase, tufA, eno, gap, groL, msmX, pckA, ppdK, and spoIVCA. Whole-genome FASTA files were annotated with Prokka (v[1.14.5]) using default parameters to produce GFF3 feature annotations (genes, rRNA/tRNA, CDS, and product fields). Unmapped reads from the alignment to the host genome were mapped to the bacterial references using Bowtie2 (v[2.5.1]) with default parameters after building indexes via bowtie2-build. Genome-wide mapping summaries were visualized with pycirclize (v[1.6.0]) as Circos-style plots. Tracks included (i) annotated ribosomal RNA/protein genes from the Prokka GFF, (ii) optional GC/AT content, and (iii) 1 kb binned coverage. Values used for plotting were clipped to the predefined display range to avoid axis boundary artefacts. The per-base depth was computed from sorted BAMs with samtools depth -aa, then aggregated into non-overlapping 1 kb windows (mean per window) and written as bedGraph (chrom, start, end, mean). Using these bins (midpoints as genomic positions), we optionally smoothed coverage (window = 1 bin; none) and called peaks with scipy.signal.find_peaks (prominence = 0.2 × 10th percentile of positive bins; minimum distance = 5 bins ≈ 5 kb). Prokka GFF3 annotations (gene/CDS/rRNA/tRNA) were parsed, and multi-segment features with the same name were merged (min start, max end). Each peak was labeled overlap if its bin intersected any feature (all overlaps recorded, one representative chosen) or nearest otherwise (nearest feature and distance reported).

Spatial autocorrelation analysis
Moran's I was calculated for the major genera (abundance > 0.01%) using the Moran function from the Python library pysal. Spatial weights were generated using the -nearest neighbors (KNN) matrix (=4) from the weights module in pysal. For genera with a Moran's I p-value < 0.05, Ripley's H was subsequently derived using the formula written on the manuscript.

Spatial co-localization analysis
To assess the spatial co-localization of bacterial genera in the Stereo-seq datasets, we applied the Smoothie package using default parameters for the Stereo-seq platform29. Briefly, bacterial spatial data classified at the genus level by Kraken2 were spatially smoothed (20 µm Gaussian kernel), and pairwise correlations were computed among all resulting bacterial signal surfaces to quantify co-localization patterns across the tissue area.

Spatial clustering of microbial signal (HDBSCAN31) and contouring
To assess the spatial clustering characteristics of a given bacterial taxa, we subset spots with nonzero counts and used their spatial coordinates (obsm['spatial']) as input to HDBSCAN (v[0.8.38]). To choose hyperparameters, we performed a grid search over min_cluster_size and min_samples. For each setting we fit HDBSCAN, removed noise labels (-1) and clusters with size < min_cluster_size, and computed the silhouette score on the remaining points. The best setting was selected by the maximum silhouette score.HDBSCAN was called with Euclidean distance and default options unless stated; noise points (label = −1) were excluded from silhouette computation. Silhouette scores were computed with scikit-learn (v[1.3.1]).

Boundary detection
The microscope data was saved in grayscale and then averaged using the OpenCV blur function with a kernel size 100 µm. After that, the data was binarized with a threshold of 80 for normal tissue and 100 for cancer tissue. Finally, boundaries were extracted using the OpenCV findContours function.

Code associated with this work can be found at https://github.com/ntekasi/microSTRS.

For manuscripts utilizing custom algorithms or software that are central to the research but not yet described in published literature, software must be made available to editors and reviewers. We strongly encourage code deposition in a community repository (e.g. GitHub). See the Nature Portfolio guidelines for submitting code & software for further information.

Data

Policy information about availability of data

All manuscripts must include a data availability statement. This statement should provide the following information, where applicable:

- Accession codes, unique identifiers, or web links for publicly available datasets
- A description of any restrictions on data availability
- For clinical datasets or third party data, please ensure that the statement adheres to our policy

Data will be made available upon publication under GEO accession numbers; GSE276866 for the low-resolution datasets, GSE277196, GSE277197, GSE308507, GSE316608 and GSE316962 for the high-resolution datasets, and GSE316869 for the bulk RNA-seq datasets.

Research involving human participants, their data, or biological material

Policy information about studies with human participants or human data. See also policy information about sex, gender (identity/presentation), and sexual orientation and race, ethnicity and racism.

Reporting on sex and gender	*Use the terms sex (biological attribute) and gender (shaped by social and cultural circumstances) carefully in order to avoid confusing both terms. Indicate if findings apply to only one sex or gender; describe whether sex and gender were considered in study design; whether sex and/or gender was determined based on self-reporting or assigned and methods used. Provide in the source data disaggregated sex and gender data, where this information has been collected, and if consent has been obtained for sharing of individual-level data; provide overall numbers in this Reporting Summary. Please state if this information has not been collected. Report sex- and gender-based analyses where performed, justify reasons for lack of sex- and gender-based analysis.*
Reporting on race, ethnicity, or [illegible]	*Please specify the socially constructed or socially relevant categorization variable(s) used in your manuscript and explain why*

Reporting on race, ethnicity, or other socially relevant groupings	*they were used. Please note that such variables should not be used as proxies for other socially constructed/relevant variables (for example, race or ethnicity should not be used as a proxy for socioeconomic status).*<br>*Provide clear definitions of the relevant terms used, how they were provided (by the participants/respondents, the researchers, or third parties), and the method(s) used to classify people into the different categories (e.g. self-report, census or administrative data, social media data, etc.)*<br>*Please provide details about how you controlled for confounding variables in your analyses.*
Population characteristics	*Describe the covariate-relevant population characteristics of the human research participants (e.g. age, genotypic information, past and current diagnosis and treatment categories). If you filled out the behavioural & social sciences study design questions and have nothing to add here, write "See above."*
Recruitment	*Describe how participants were recruited. Outline any potential self-selection bias or other biases that may be present and how these are likely to impact results.*
Ethics oversight	*Identify the organization(s) that approved the study protocol.*

Note that full information on the approval of the study protocol must also be provided in the manuscript.

Field-specific reporting

Please select the one below that is the best fit for your research. If you are not sure, read the appropriate sections before making your selection.

☒ Life sciences ☐ Behavioural & social sciences ☐ Ecological, evolutionary & environmental sciences

For a reference copy of the document with all sections, see nature.com/documents/nr-reporting-summary-flat.pdf

Life sciences study design

All studies must disclose on these points even when the disclosure is negative.

Sample size	One sample per condition was collected using the protocol in this manuscript, but eight total samples were collected for low-resolution analysis and three more samples were collected for high-resolution analysis.
Data exclusions	For downstream analysis, data from outside the tissues areas were excluded when noted.
Replication	We processed 18 samples in total using the method described in this manuscript, and assessed quality of each sample to ensure the repeatability of Spatial Total RNA-Sequencing.
Randomization	NA
Blinding	NA

Reporting for specific materials, systems and methods

We require information from authors about some types of materials, experimental systems and methods used in many studies. Here, indicate whether each material, system or method listed is relevant to your study. If you are not sure if a list item applies to your research, read the appropriate section before selecting a response.

Materials & experimental systems

n/a	Involved in the study	
☒	☐	Antibodies
☒	☐	Eukaryotic cell lines
☒	☐	Palaeontology and archaeology
☐	☒	Animals and other organisms
☒	☐	Clinical data
☒	☐	Dual use research of concern
☒	☐	Plants

Methods

n/a	Involved in the study	
☒	☐	ChIP-seq
☒	☐	Flow cytometry
☒	☐	MRI-based neuroimaging

Animals and other research organisms

Policy information about studies involving animals; ARRIVE guidelines recommended for reporting animal research, and Sex and Gender in Research

Laboratory animals	All animal protocols were approved by the Cornell University Institutional Animal Care and Use Committee (IACUC), and experiments were performed in compliance with institutional guidelines. C57BL/6-ApcMin/+/J mice were used for the spatial transcriptomics experiments. All mice (C57BL/6-ApcMin/+/J and C57BL/6-Wild type) were maintained at the barrier mouse facility at Weill Hall of

	Cornell University. ApcMin/+ and wild-type mice were initially ordered from Jackson Laboratory and then bred in the barrier facility. The ApcMin/+ mice used in these experiments have a chemically induced transversion point mutation at nucleotide 2549, resulting in a stop codon at codon 850, truncating the APC protein.
Wild animals	No wild animals were used in this study.
Reporting on sex	Both male and female mice were used, and their precise age was noted.
Field-collected samples	NA
Ethics oversight	The Cornell University Institutional Animal Care and Use Committee (IACUC) approved all animal protocols, and experiments were performed in compliance with its institutional guidelines (protocol number : IACUC 2016-0088).

Note that full information on the approval of the study protocol must also be provided in the manuscript.

Plants

Seed stocks	NA
Novel plant genotypes	NA
Authentication	NA
