# Supplementary material for: Spatial transcriptomics maps host–gut microbiome biogeography at high resolution
Source: Nat Microbiol. 2026 Mar 6;11(5):1193–204. doi: 10.1038/s41564-026-02286-7 (PMC13171632; doi:10.1038/s41564-026-02286-7)
Supplement: Supplementary file 1 — Supplementary Figs. 1–20. [file 41564_2026_2286_MOESM1_ESM.pdf]

nature microbiology

Supplementary information https://doi.org/10.1038/s41564-026-02286-7

Spatial transcriptomics maps host–gut microbiome biogeography at high resolution

In the format provided by the authors and unedited

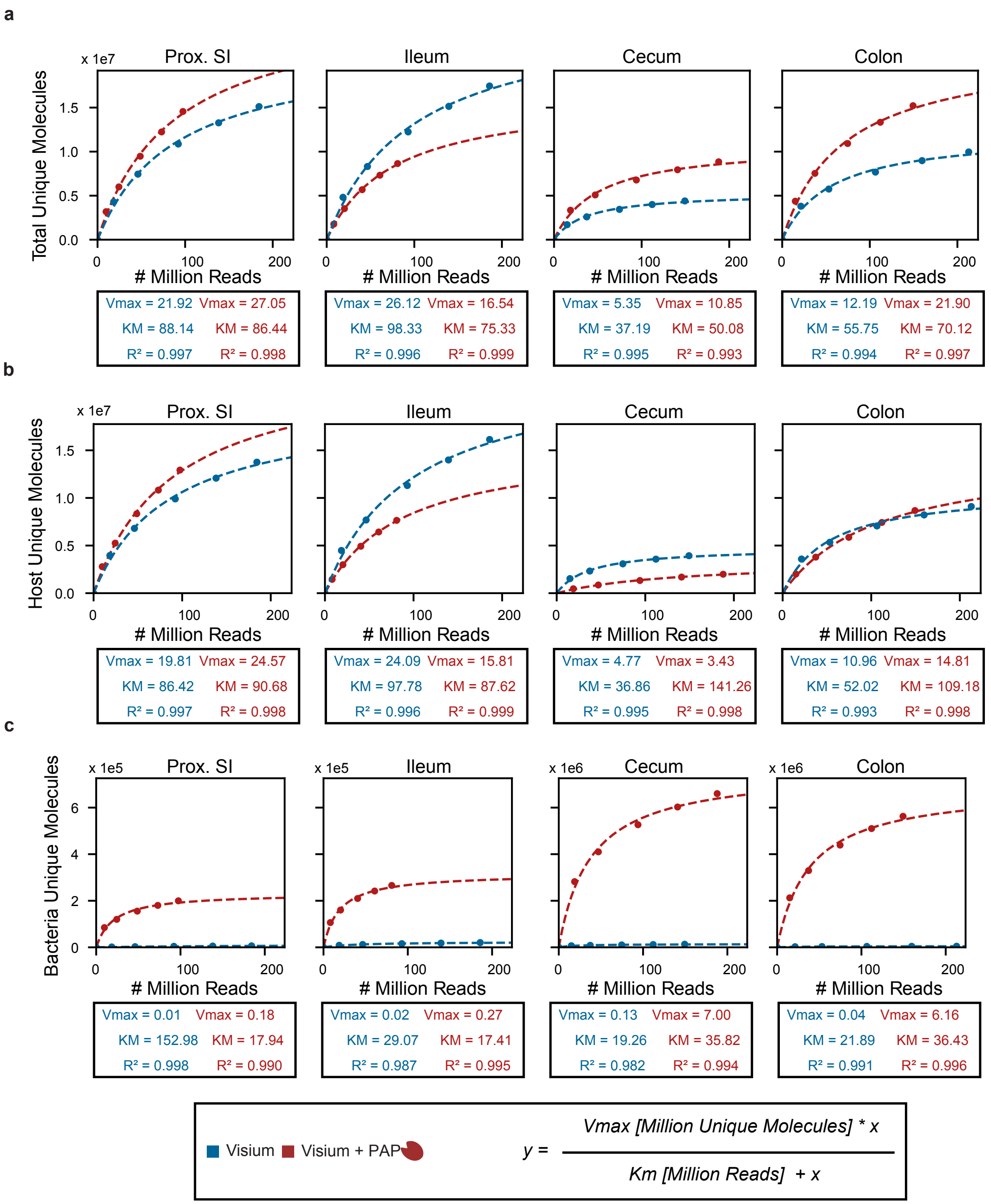

Supplementary Figure 1. Rarefaction analysis of unique-molecule yield versus sequencing depth for paired Visium (blue) and Visium + *in situ* polyadenylation (PAP; red) libraries across four intestinal sites: proximal small intestine (Prox. SI), ileum , cecum , and colon. (a) Total unique molecules, (b) host-aligned unique molecules, and (c) bacteria-classified unique molecules, each plotted as a function of input depth (million reads). Points show values from read subsampling. Colored dashed curves are Michaelis–Menten fits with Vmax (Million Unique molecules), Km (Million Reads), and goodness-of-fit (R^2) reported under each plot.

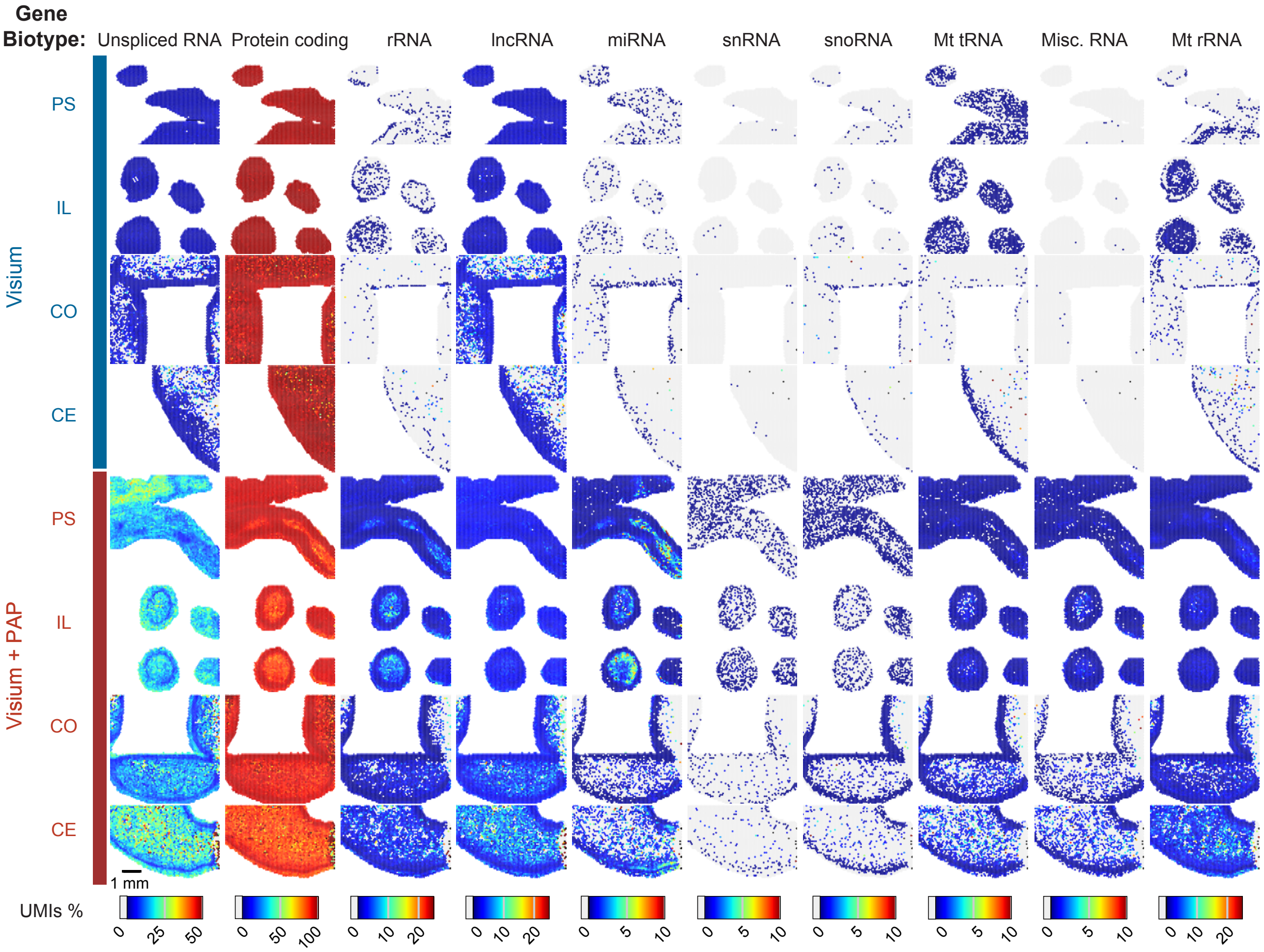

Supplementary Figure 2. RNA biotype distributions for the paired Visium and STRS experiments. Spatial maps showing the patterns of the biotypes across the paired experiments.

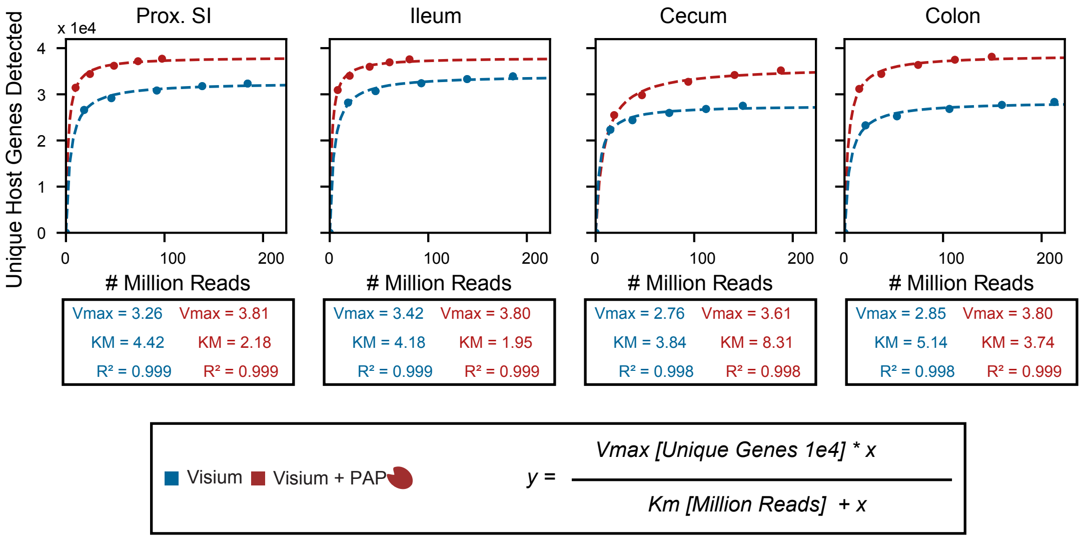

Supplementary Figure 3. Rarefaction analysis of unique host genes versus sequencing depth for paired Visium (blue) and Visium + *in situ* polyadenylation (PAP; red) libraries across four intestinal sites: proximal small intestine (Prox. SI), ileum, cecum, and colon. Points show values from read subsampling. Colored dashed curves are Michaelis–Menten fits with Vmax (Million unique genes), Km (Million Reads), and goodness-of-fit (R^2) reported under each plot.

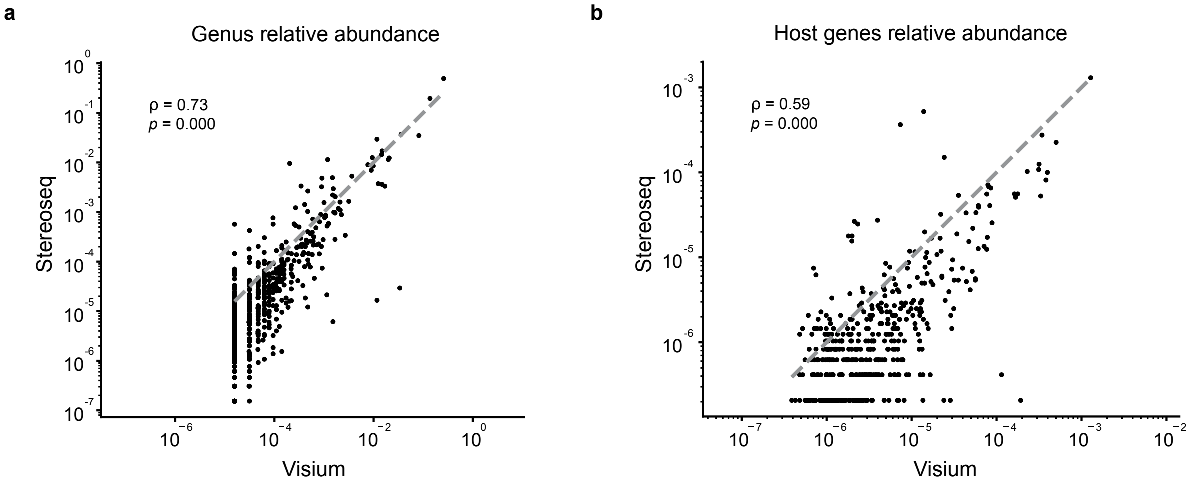

Supplementary Figure 4. Comparison between low resolution platform Visium and high-resolution platform Stereoseq. (a) Genus-level relative abundance measured on the Visium (*in situ* polyadenylation) and high resolution Stereoseq platform (*in situ* polyadenylation). (b) Host gene relative abundance measured on the Visium (*in situ* polyadenylation) and high resolution Stereoseq platform (*in situ* polyadenylation). Speaman's ρ and two-sided p-values are shown.

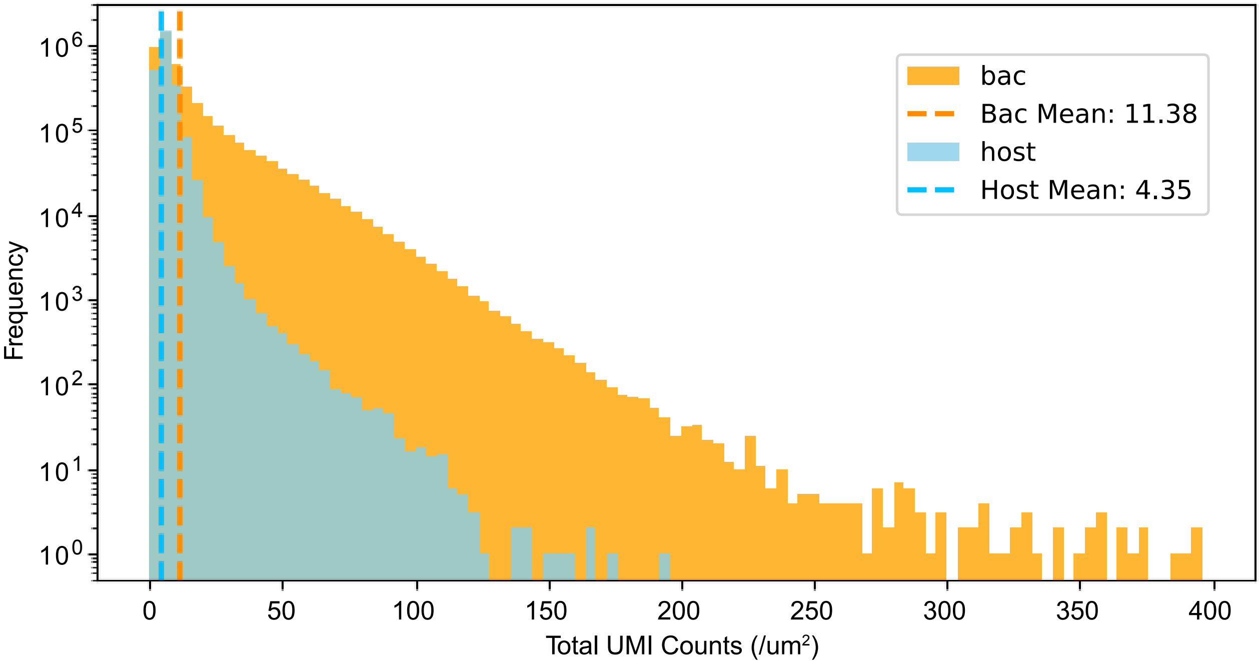

Supplementary Figure 5. The frequency distribution and mean of UMI counts per μm^2 across the whole tissue and lumen areas.

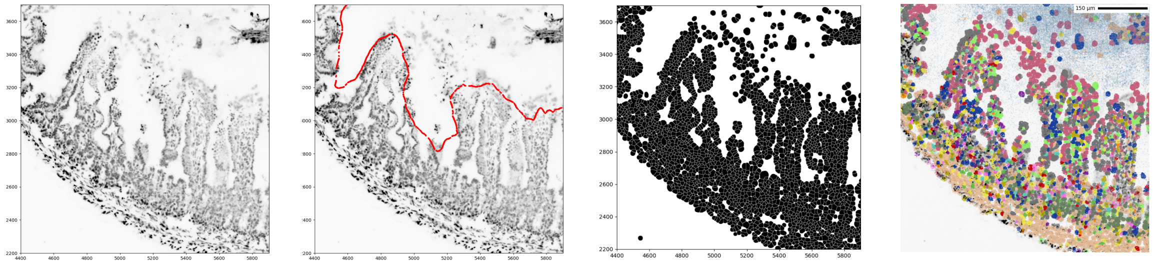

Supplementary Figure 6. Cell segmentation of the Stereo-seq + PAP processed ileum cross-section. From left to right: Close-up of the nucleus-stained fluorescent image of an ileum cross-section, Host boundary overlay with red line, cell segmentation results of the close-up, cell type spatial maps after deconvolution.

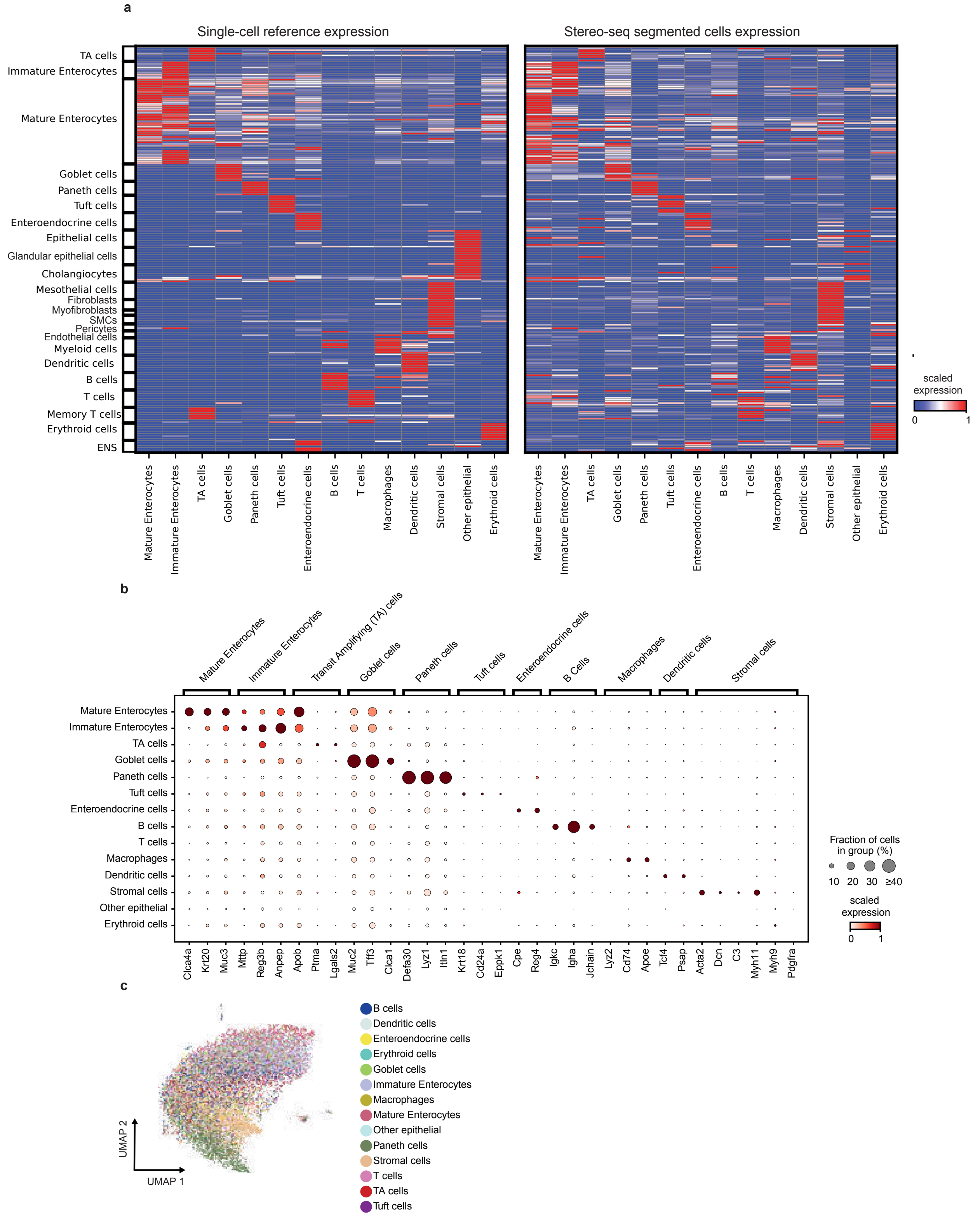

Supplementary Figure 7. Cell type deconvolution validation. (a) Heatmaps of canonical markers' gene expression for the single-cell reference used for the deconvolution (left) and the cell-binned spatial transcriptomics data (right). (b) Dot-plot of scaled gene expression for various canonical markers per cell type for the ileum dataset processed with Stereo-seq + PAP. The cicle size denotes the fraction of cells per deconvolution assignment expressing the gene and color denotes the scaled gene expression in the group. (c) UMAP projection of the cell-binned spatial data colored by cell type deconvolution assignment.

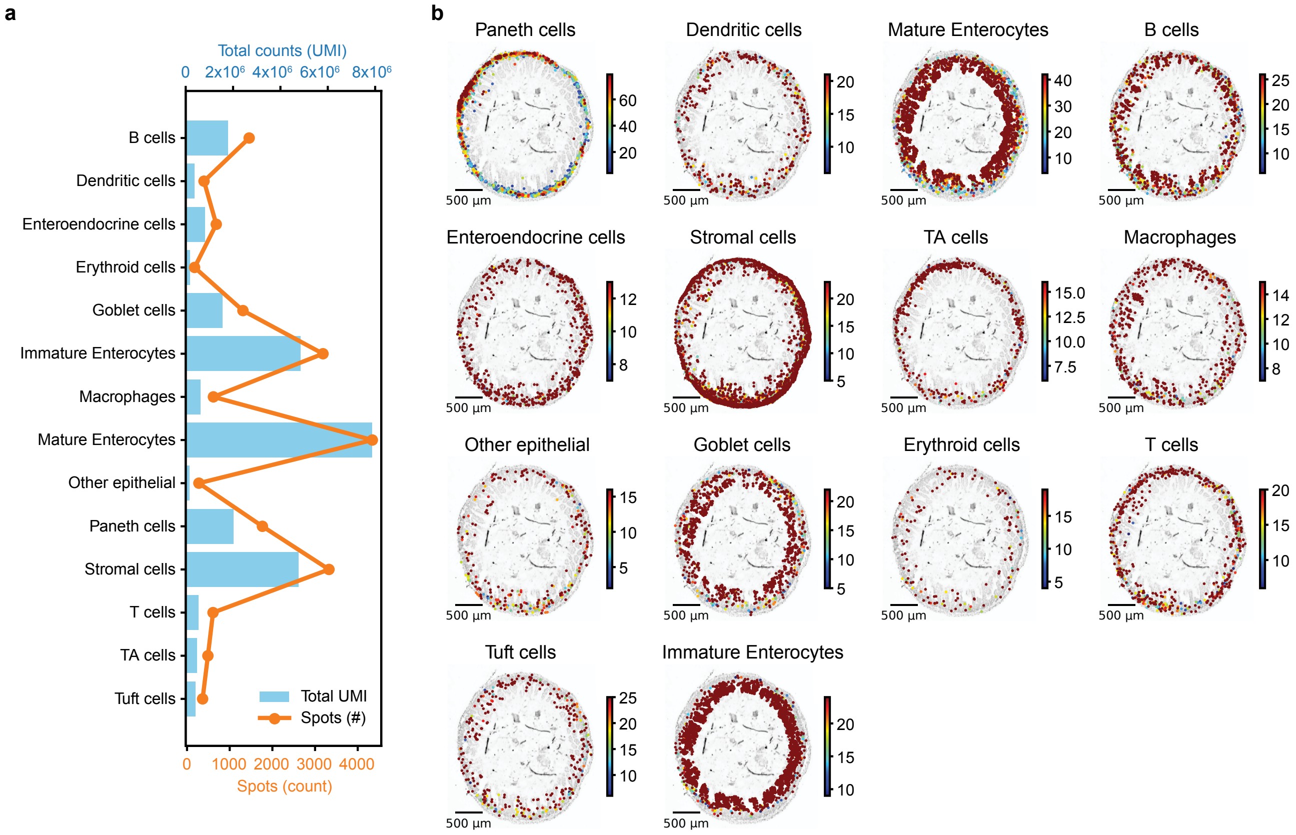

Supplementary Figure 8. Cell type analysis. (a) The total number of cells and RNA molecules for each cell type. (b) Spatial maps of each cell type. Color scale represents total gene expression for each cell within the plotted cell type.

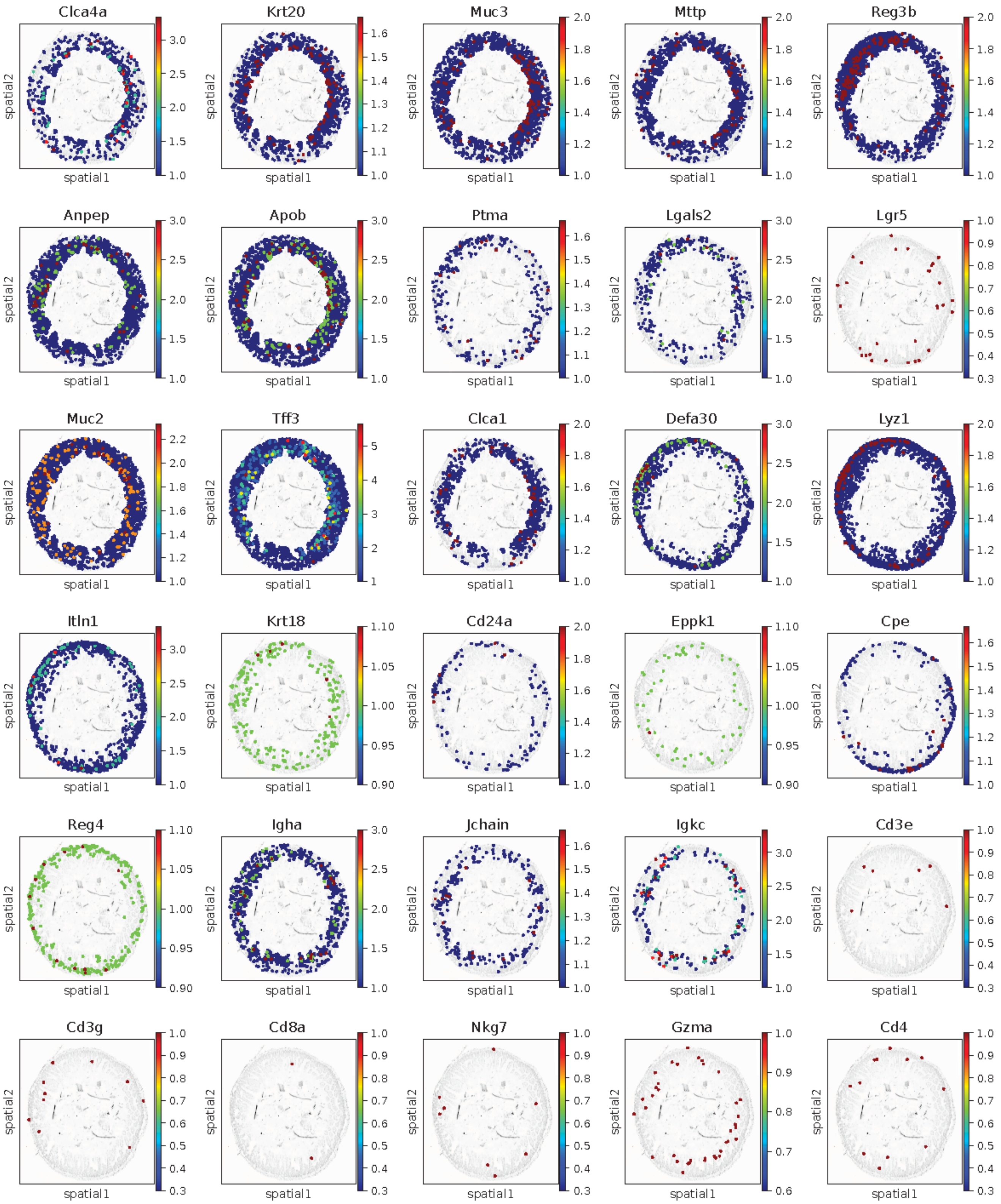
Clca4a
Krt20
Muc3
Mttp
Reg3b
Anpep
Apob
Ptma
Lgals2
Lgr5
Muc2
Tff3
Clca1
Defa30
Lyz1
Itln1
Krt18
Cd24a
Eppk1
Cpe
Reg4
Igha
Jchain
Igkc
Cd3e
Cd3g
Cd8a
Nkg7
Gzma
Cd4
spatial1
spatial2

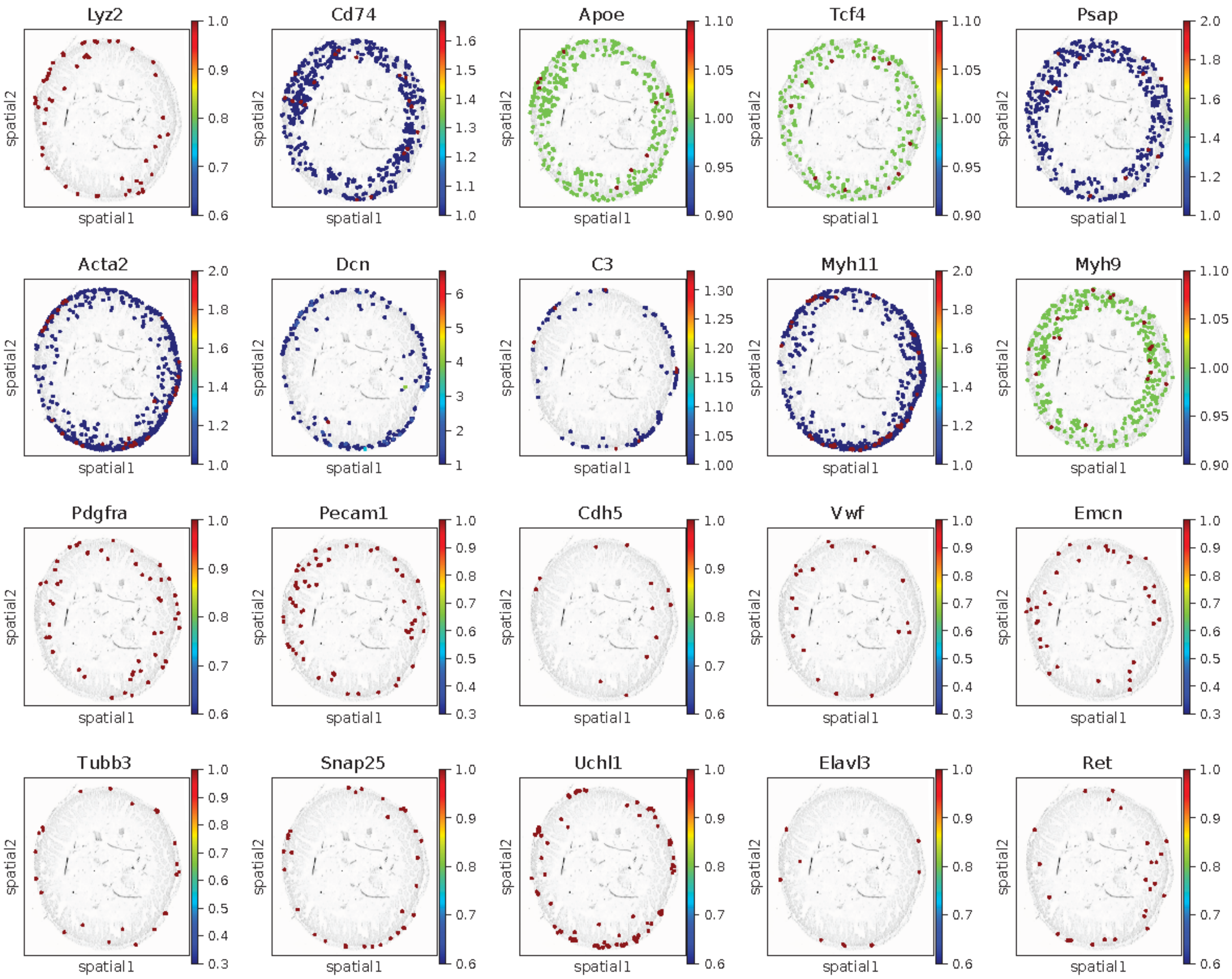

Supplementary Figure 9. Spatial maps of raw gene expression for various intestinal canonical cell type markers.

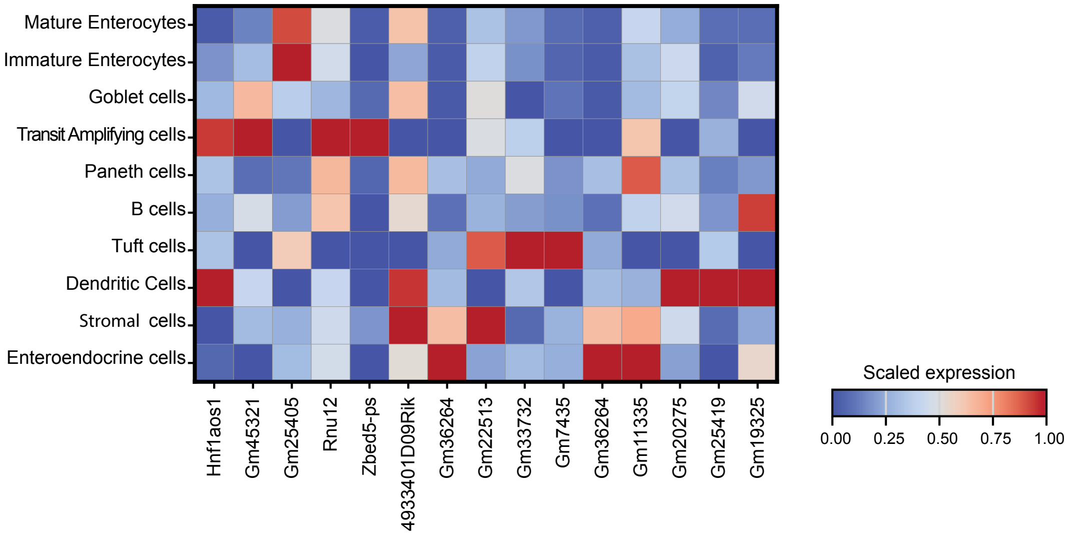

Supplementary Figure 10. Heatmap of the normalized amount of non-coding genes for each cell type. We used min-max normalization to ensure that the smallest value for each gene was set to 0 and the largest value was set to 1.

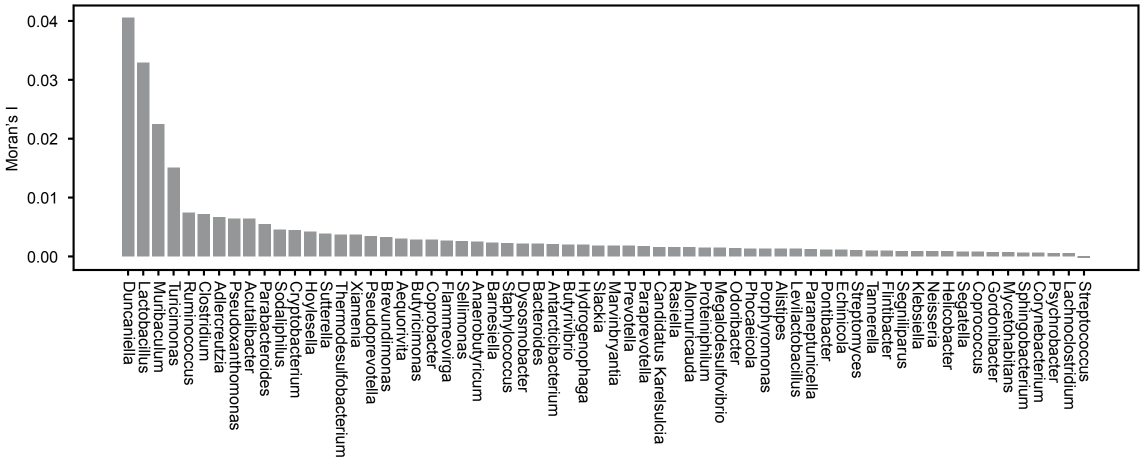

Supplementary Figure 11. Spatial autocorrelation and correlation analysis in genera. Moran's I scores of major genera (>0.01% total bacterial counts, $p < 0.05$).

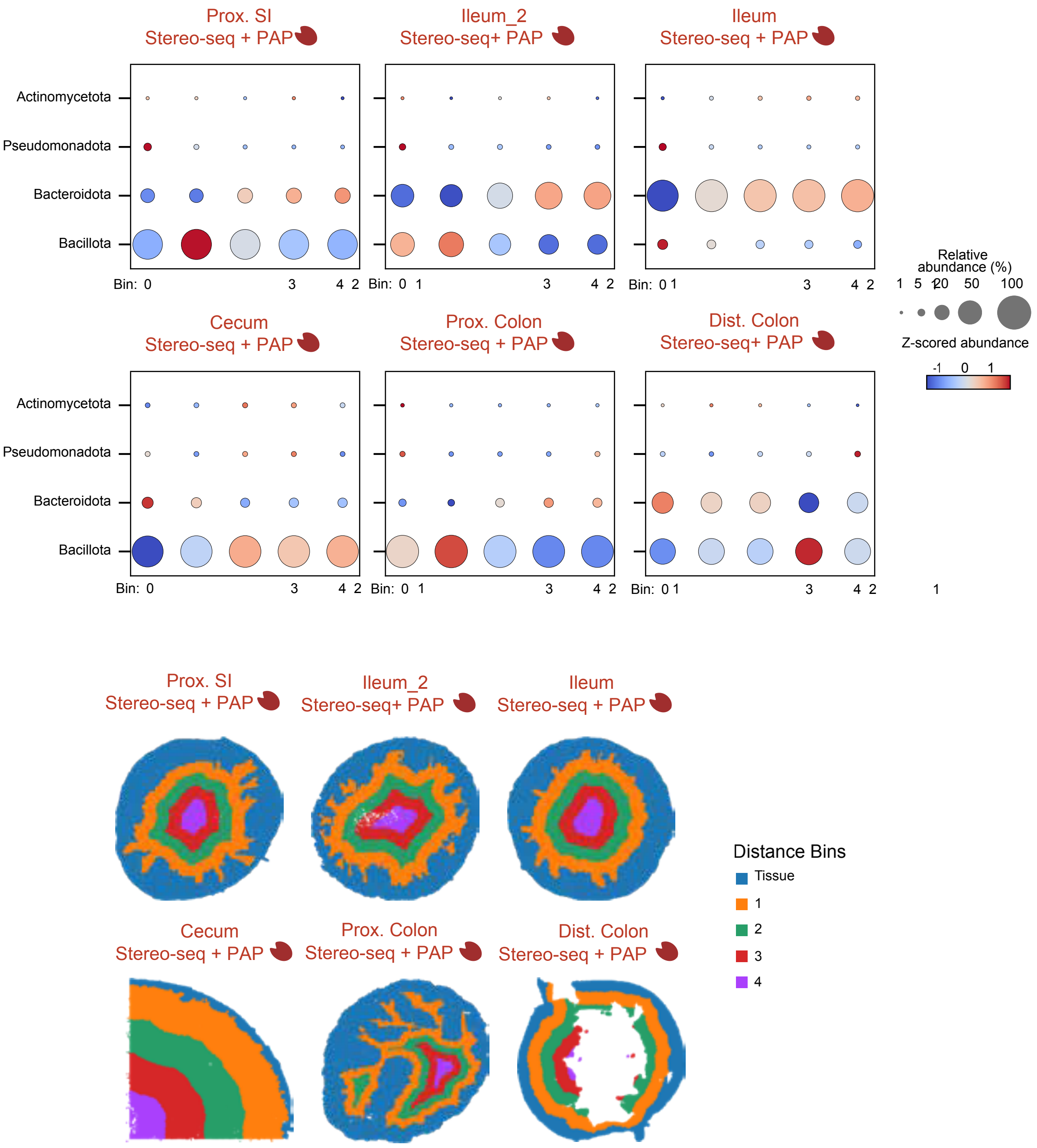

Supplementary Figure 12. Phylum-level microbial abundance patterns across 5 sites of the murine intestine. (a) Relative abundance of microbial taxa along the transverse axis (from tissue to lumen) at the phylum level for five GI locations. Dot plots for each location display the relative abundance of four major phyla, divided into 5 spot-distance bins from the tissue. Dot size represents the relative abundance percentage, and the color indicates the relative abundance z-score across bins. (b) Maps showing the spatial bins used for the relative abundance calculation.

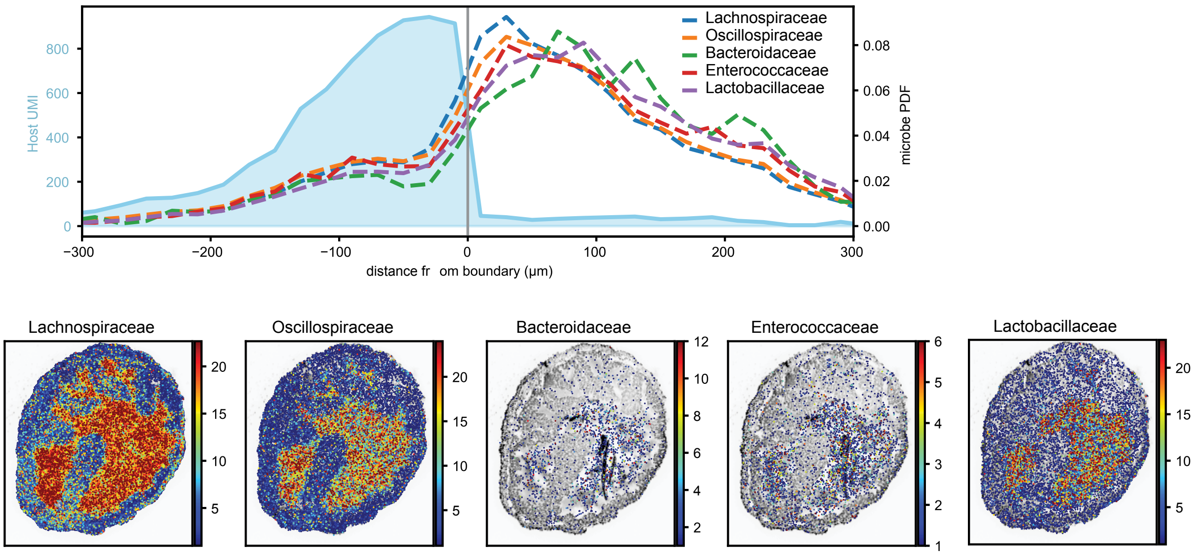

Supplementary Figure 13 Spatial localization pattern of microbial familes in the Proximal Colon of a female mouse. Probability density function of select microbial families relative to the tissue boundary and host detected unique molecules expressed as Host UMI. Spatial abundance maps for the 5 microbial families.

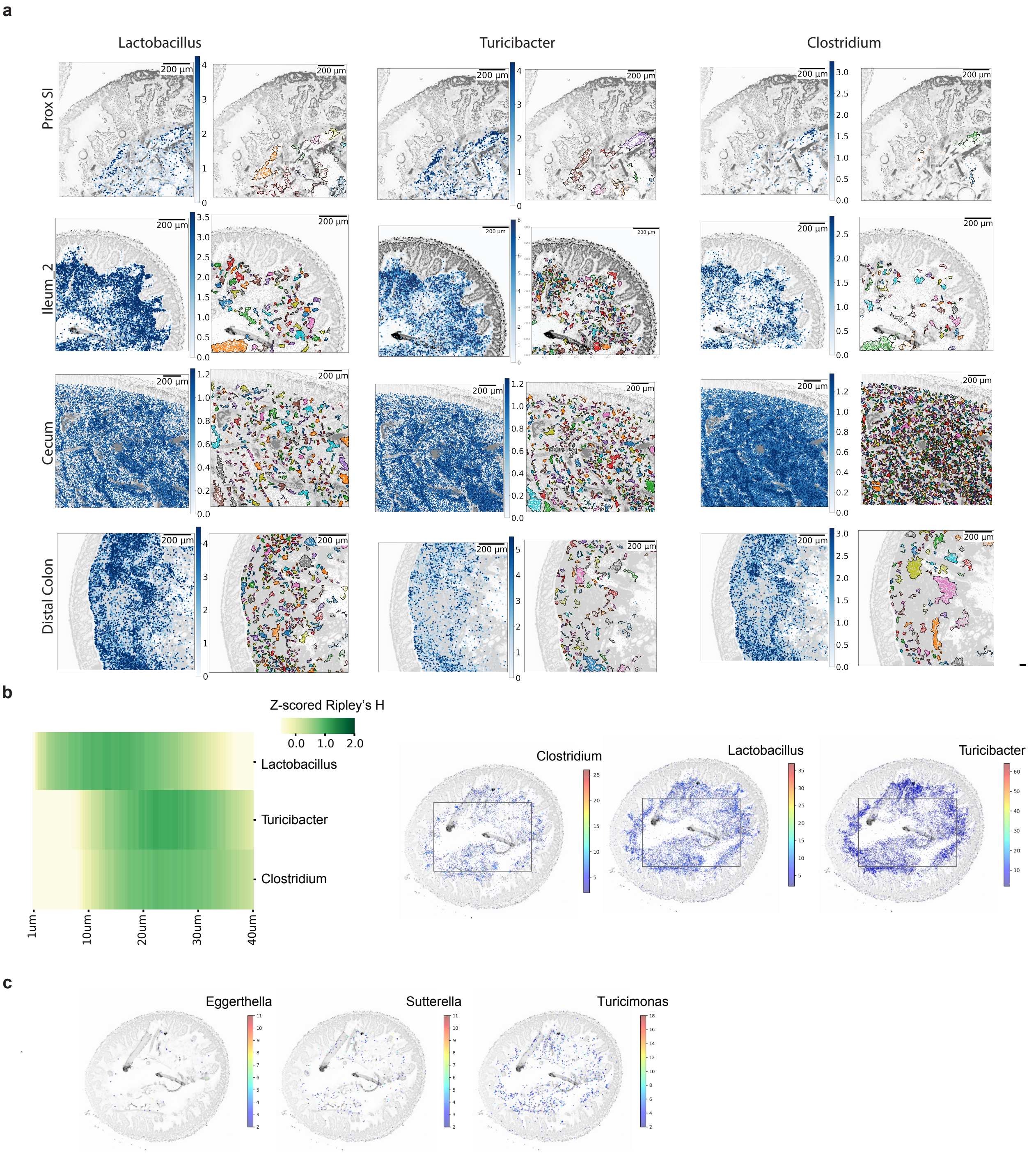

Supplementary Figure 14. (a) Bacterial genera signal and colonies identified with HDBSCAN in four intestinal sites from the same mouse. (b) Z-scored Ripley's H score (left) and spatial maps of corresponding genera (right). The square indicates the region used for the calculation of Ripley's H. (c) Spatial maps of genera in Figure 3.

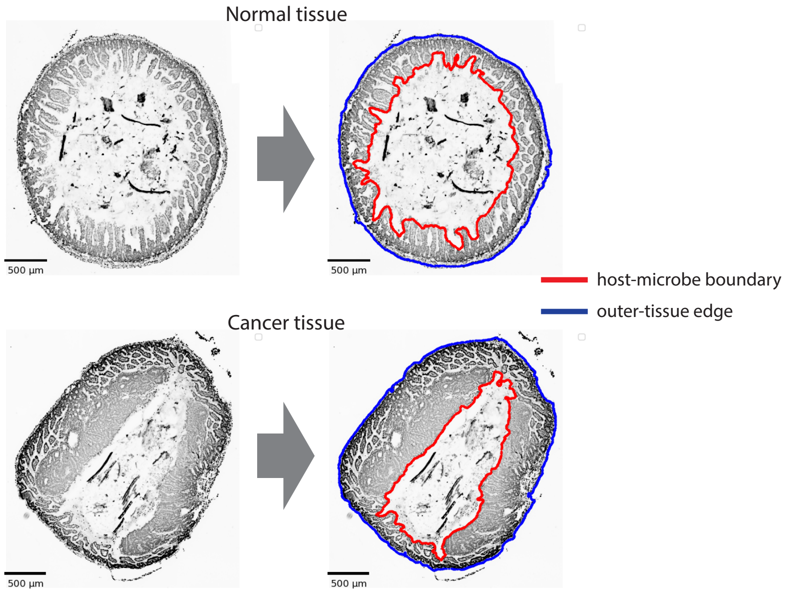

Supplementary Figure 15. Defined boundaries for lumen, tissue area based on the microscope images. After average smoothing in 100um, image data were binarized to detect the boundaries for both host-microbe boundaries and out-tissue edges in cancer and normal samples.

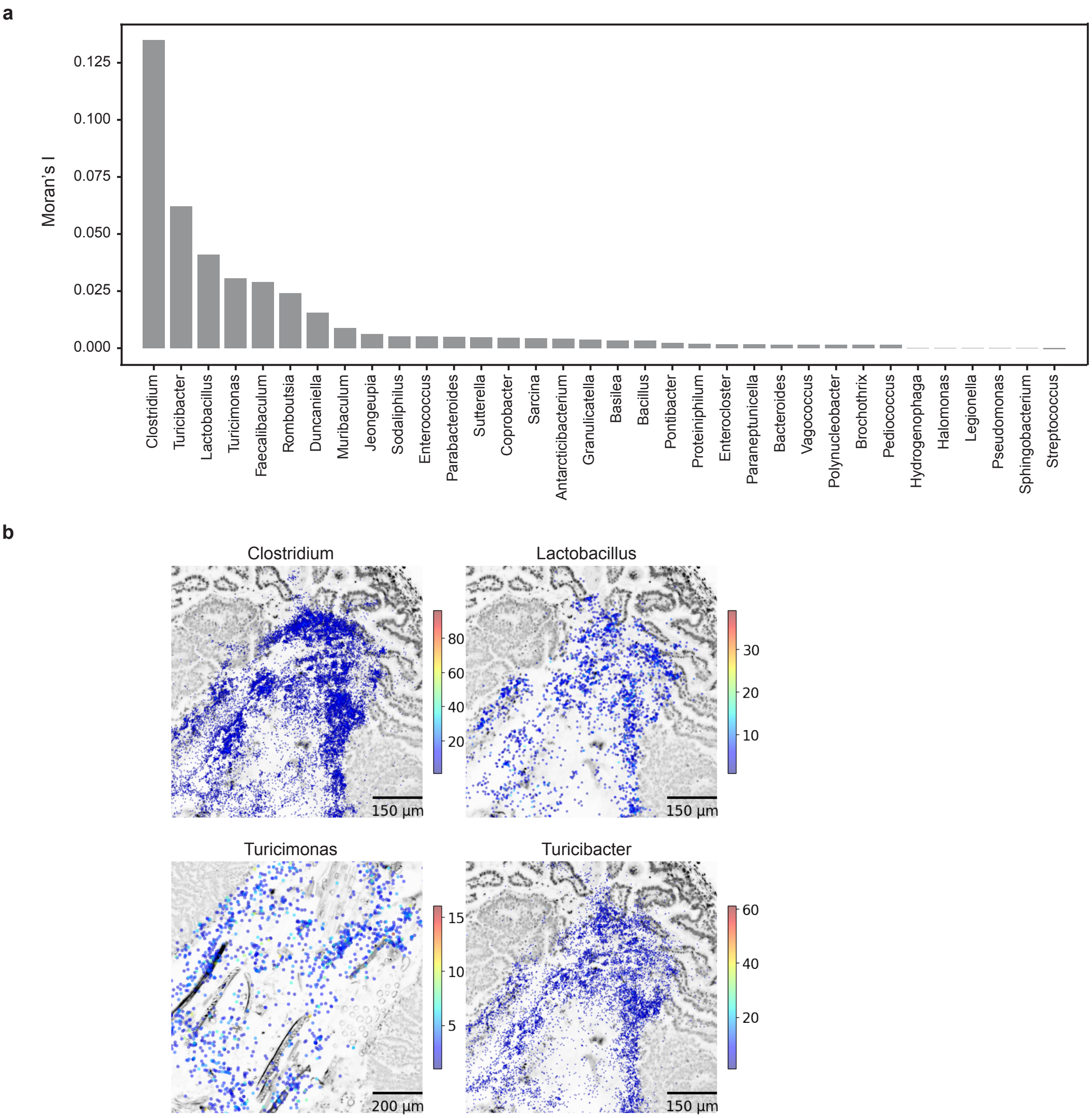

Supplementary Figure 16. Autocorrelation in cancer section. (a) Moran's I scores of major genera (>0.01% total bacterial counts, $p < 0.05$). (b) Enlarged spatial mapping image of selected genera.

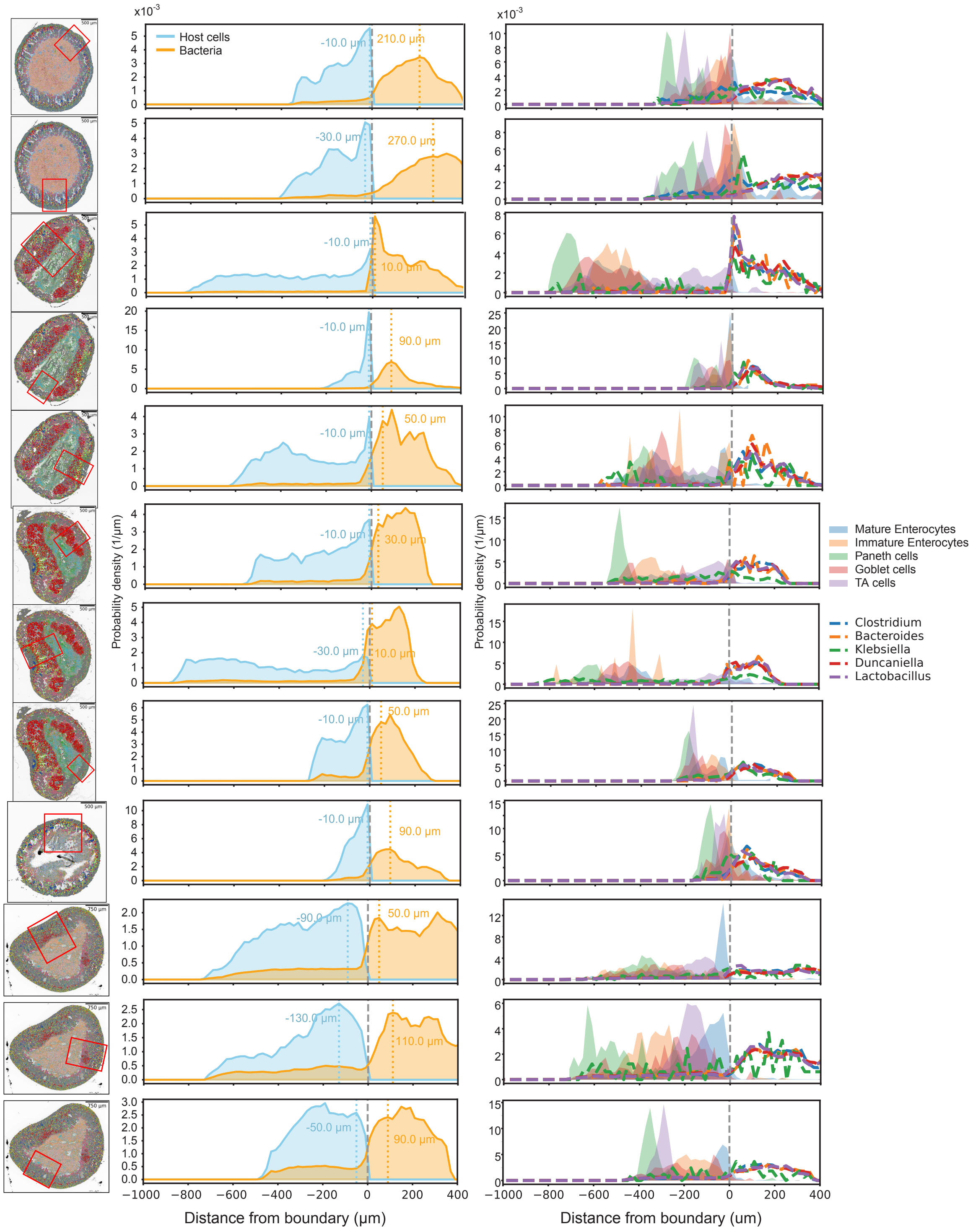

Host cells
Bacteria
Probability density (1/μm)
Distance from boundary (μm)
Distance from boundary (um)
Mature Enterocytes
Immature Enterocytes
Paneth cells
Goblet cells
TA cells
Clostridium
Bacteroides
Klebsiella
Duncaniella
Lactobacillus

Supplementary Figure 17. Host and microbiome derived molecules as a function of the distance to the host-microbiome interface in tumor and healthy ileum cross-sections. (left) Spatial cell type and microbiome maps of healthy and tumor-bearing sections, the red rectangle denoted the region of interest (ROI). (middle) Host-cell derived (blue) and microbiome-derived (orange) molecular signal as identified in the corresponding ROI, the dashed line denotes the boundary. The distance of the closest to the boundary local maxima for host and microbiome signal is marked with blue for host and orange for microbiome. (right) Per cell-type and per genera binned molecular signal for select cell-types and microbial genera.

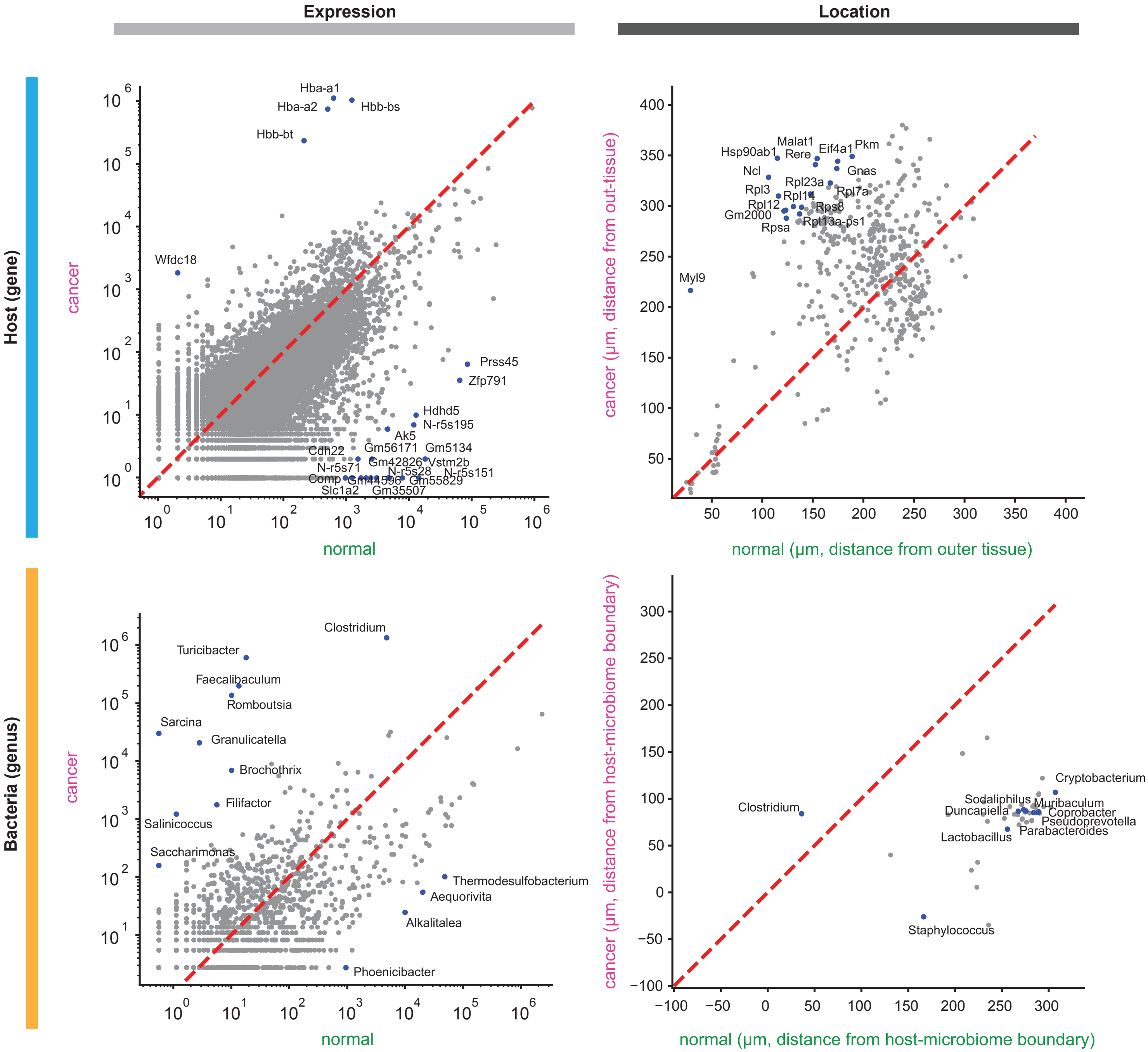

Supplementary Figure 18. Difference in location of the host genes and microbes between normal and cancer sections. Gene abundance of normal and cancer sections(left-above), and genus level abundance in normal and cancer sections (left-below). Median distance of genes from out-tissue area (right-above), and genera from host-microbe boundary (right-below).

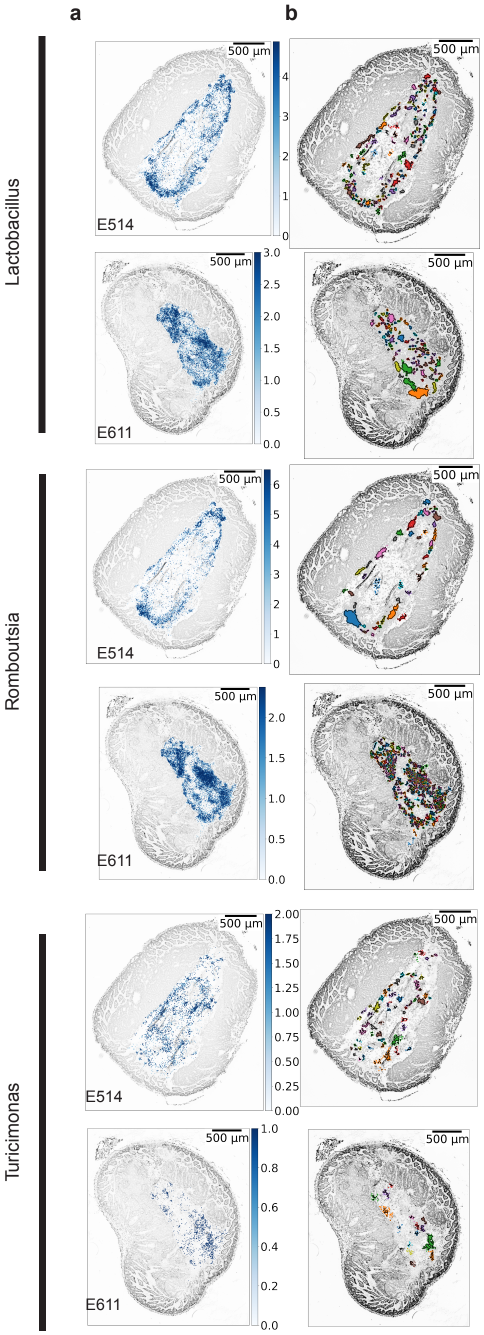

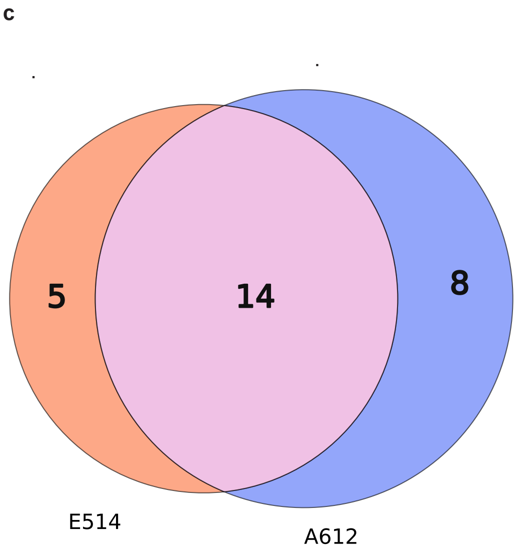

d

Bacillus
Clostridium
Duncaniella
Enterococcus
Faecalibaculum
Lactobacillus
Muribaculum
Parabacteroides
Romboutsia
Sodaliphilus
Staphylococcus
Stenotrophomonas
Turicibacter
Turicimonas

Supplementary Figure 19. Bacterial colonies in tumour sections from the same Apc-deficient mouse. (a) Spatial distribution of the colony-forming genera *Lactobacillus*, *Romboutsia* and *Turicimonas*. (b) Colony identification with HDBSCAN. (c) Venn diagram showing the bacterial genera with spatial autocorrelation in the two tumour sections (Moran's I > 0, FDR-adjusted $p<0.05$). (d) List of shared colony-forming genera as identified by the Moran's I test.

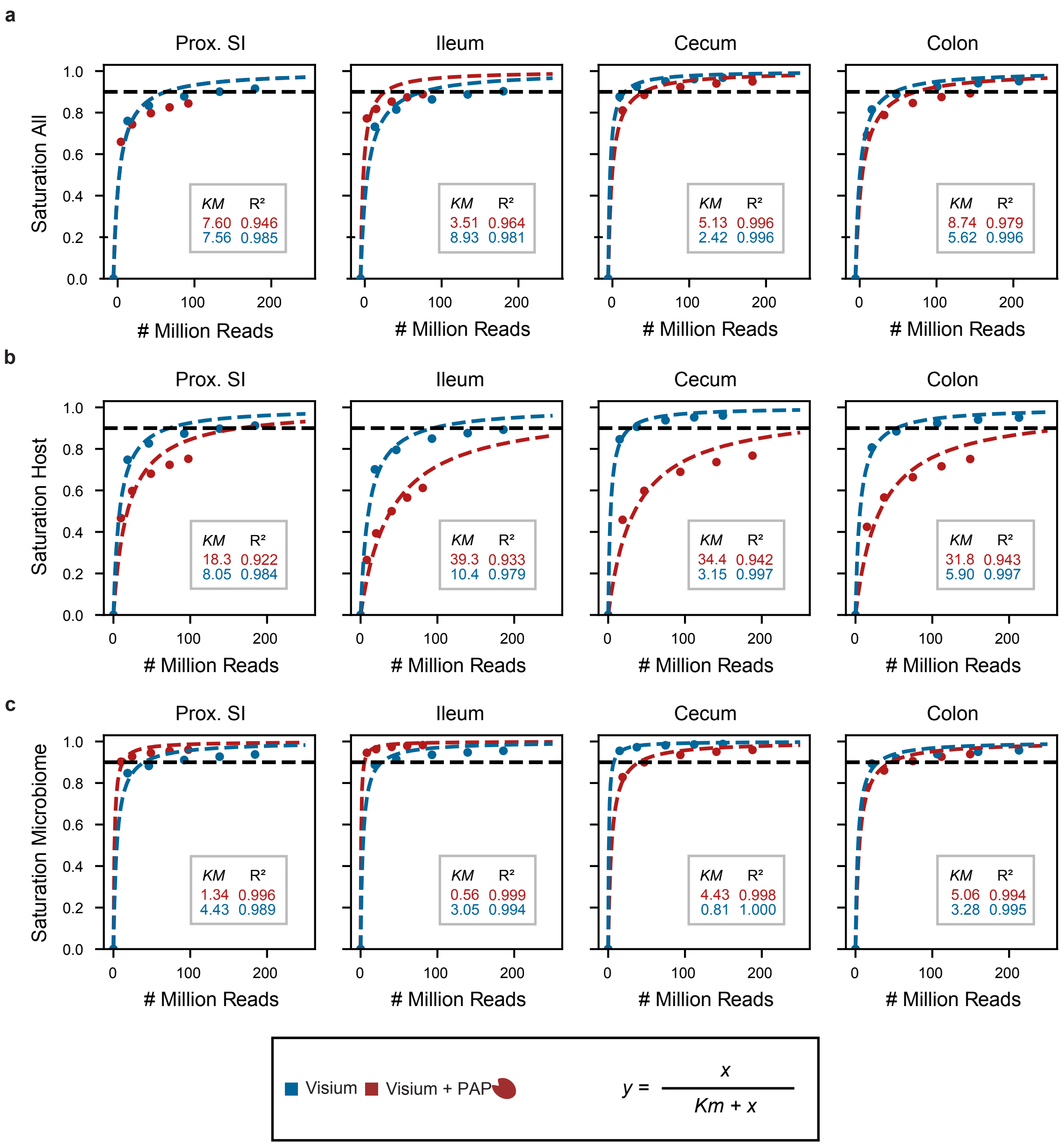

Supplementary Figure 20. Empirical sequencing saturation curves for paired Visium (blue) and Visium + *in situ* polyadenylation (PAP; red) libraries across four intestinal sites: proximal small intestine, ileum, cecum, and colon. (a) Saturation of the total library, (b) saturation of host-aligned molecules, and (c) saturation of microbiome-classified molecules, each calculated across sequencing depths. Colored dashed lines indicate Michaelis–Menten fits, with inset values reporting the fitted constant KM and the goodness of fit (R^2). The horizontal black dashed line marks 0.9 saturation.
